# Effects of an 8-Week Active Play Intervention on Body Composition and Fundamental Motor Skills in Preschool Children †

**DOI:** 10.3390/children11101173

**Published:** 2024-09-26

**Authors:** Katherine E. Spring, Danielle Lang, Melissa M. Pangelinan, Danielle D. Wadsworth

**Affiliations:** 1 School of Kinesiology, Auburn University, Auburn, AL 36849, USA; dmc0046@auburn.edu (D.L.); mpangel@iu.edu (M.M.P.); wadswdd@auburn.edu (D.D.W.); 2 Pediatric Obesity and Health Behavior Laboratory, Division of Population and Public Health Science, Pennington Biomedical Research Center, Baton Rouge, LA 70808, USA; 3 Research Transition Office, Center for Military Psychiatry and Neuroscience, Walter Reed Army Institute of Research, Silver Spring, MD 20910, USA; 4 School of Public Health Bloomington, Indiana University, Bloomington, IN 47405, USA

**Keywords:** development, early childhood education, physical activity, randomized control trial

## Abstract

**Objective:** Examine the effect of an 8-week teacher-guided active play intervention on preschoolers’ body composition and fundamental motor skills. **Methods:** Participants were from two local preschool centers randomly assigned to either the intervention (*n* = 25, 3.91 ± 0.53 years) or the control group (*n* = 25, 3.69 ± 0.81 years). All measures were assessed at baseline (week 0), post-intervention (weeks 9–11), and follow-up (weeks 30–33). Bioelectrical Impedance assessed body composition (fat mass (FM) and fat-free mass (FFM)). The Peabody Developmental Motor Scales, Second Edition (PDMS-2) assessed fundamental motor skills (gross motor quartile (GMQ)). **Results:** A significant Group × Time interaction for GMQ at post-intervention (*p* = 0.03), with the intervention group scoring significantly higher on GMQ. A significant main effect of Time (*p* < 0.001) indicated that GMQ increased in both groups across the 33-week period. For FM, a significant main effect of Time at both post-intervention (*p* < 0.05) and follow-up testing (*p* < 0.001) indicated that participants increased FM over the 33-week period. Lastly, there was a significant main effect of Time for FFM at post-intervention (*p* = 0.003) and follow-up (*p* < 0.001). Interestingly, there was a significant Group × Time interaction (*p* < 0.05) at follow-up testing showing that FFM increased over time but significantly more for the control group. **Conclusions:** Results indicate that active play interventions might be a successful pathway to improve gross motor skills in young children. Further research is needed to understand the effect that active play interventions have on body composition in preschoolers.

## 1. Introduction

Early childhood is considered an imperative time for physical, cognitive, and social development [1]. Unfortunately, over the last decade, there have been growing public health concerns for this age group. For example, it is estimated that around 37 million children aged 3–5 years are considered overweight [2], fundamental motor skill competence is low in young children globally [3], and few children meet the physical activity guidelines [4]. Fundamental motor skills (FMSs), which include locomotor skills (hopping running skipping, leaping), object manipulation skills (catching, kicking), and stability (balancing), are considered building blocks for later physical activity participation [5,6,7]. Because human development is complex and changes in one area of development often lead to cascading effects in other areas, it is of no surprise that motor delays in early childhood can lead to low physical activity levels throughout the lifetime [8]. Furthermore, limited evidence suggests that excessive body fat mass in preschoolers is associated with worse locomotor skills [9], and greater amounts of fat-free mass might be related to better balance and object manipulation [10]. Unfortunately, FMSs are not acquired naturally and must be learned [11]. One effective pathway to developing FMSs is guided physical activity. In fact, current evidence suggests that guided physical activity in the first four years of a child’s life is pivotal for the development of FMSs [12].

During the preschool years (3–5), children experience rapid physical, cognitive, and social development. Play is a key facilitator to development [13,14] because children are able to explore and interact with their environment while learning and gaining skills through play [15,16]. Active play, a subcategory of play [17], is a developmentally appropriate practice to increase physical activity. In childcare settings, active play is often defined as a type of physical activity focusing on teacher-led and child-directed activities that can be incorporated into the classroom (indoor) curriculum or outdoor time [18,19]. A systematic review aiming to synthesize the literature defining, describing, and methodology of active play could only identify 28 studies that met their inclusion criteria [18]. Of the 28 studies, only one was an intervention based in a childcare setting. While O’Dwyer and colleagues did not find any significant changes in physical activity following a 6-week active play intervention, they did stress the need for more active play interventions because the research is limited and a relatively under researched area [20]. To date, much of the active play literature focuses on physical activity as an outcome variable. However, a few studies have examined the effect that active play has on fundamental motor skills and body composition. Adamo et al. found that a 6-month active play intervention was effective at improving locomotor skills in Canadian preschoolers [21]. Roach and colleagues found that preschoolers enrolled in an 8-week active play intervention saw significant increases in FMSs compared to those in the skill station group [22]. In Australia, a health initiative called “*Munch & Move*” was found to be effective at improving FMSs after 4 months [23]. However, this study did not include measures of body composition. Currently, we could only identify a single active play intervention that included body composition as an outcome variable. Goldfield and colleagues found that 1-month active play intervention significantly improved body fat percentage and fat mass [24].

Based on the limited research, it can be inferred that active play interventions can be a successful path to improving physical activity, and possibly FMSs and body composition. Unfortunately, there have only been a handful of studies that examined the effects active play has on FMSs, and far fewer on body composition. Considering that much of the literature in preschoolers relies heavily on body mass index (BMI), it is unsurprising that few active play interventions have examined the effects on body composition. However, a recent systematic review has called for preschool-aged studies to include measures of body composition, as well as anthropometric measures, for researchers to have a better idea of how our interventions impact a child’s development and health [25]. Therefore, the purpose of this study was to examine the effect of an 8-week teacher-guided active play intervention on preschoolers’ fundamental motor skills and body composition. Researchers hypothesized that children who received the 8-week active play intervention would exhibit significant increases in FMSs, and fat-free mass (FFM) compared to the control group.

## 2. Materials and Methods

Participants were recruited from two local preschool centers in the southeastern region of the United States. These centers provide all-day childcare services for 6-week-old infants through 4-year-old preschoolers. Inclusion criteria for the present study were (1) the child was enrolled in one of the 3-, 4-,or 5 -year-old classes, and (2) parents consented to their child being a part of the study. An a priori sample size, which was calculated using G*power (Version 3.1.9.7) [26], indicated that 44 participants (22 per group) were needed to achieve 0.80 power with an alpha level of 0.05 and an effect size of 0.24. To this note, the effect size was chosen based on a meta-analysis, which found that teacher-implemented physical activity interventions have an effect size of 0.24 [27]. Due to the potential loss of participants (e.g., moving, attendance, missing measurements), researchers planned to oversample at a rate of 1.2 for a total of 53 participants (approximately 27 per group). Prior to data collection, researchers obtained written parental consent. Participants and parents were informed that participation was voluntary and that participants could choose to withdraw at any time without any consequence. Of 170 children in the 3–5-year-old classes in both centers, 52 participants returned parental consent and were eligible to participate. This study was approved by the University’s Human Subjects’ Research Institutional Review Board. The associated clinical trial number is NCT0574491.

### 2.1. Measurements

#### 2.1.1. General Procedures

**Figure 1:**
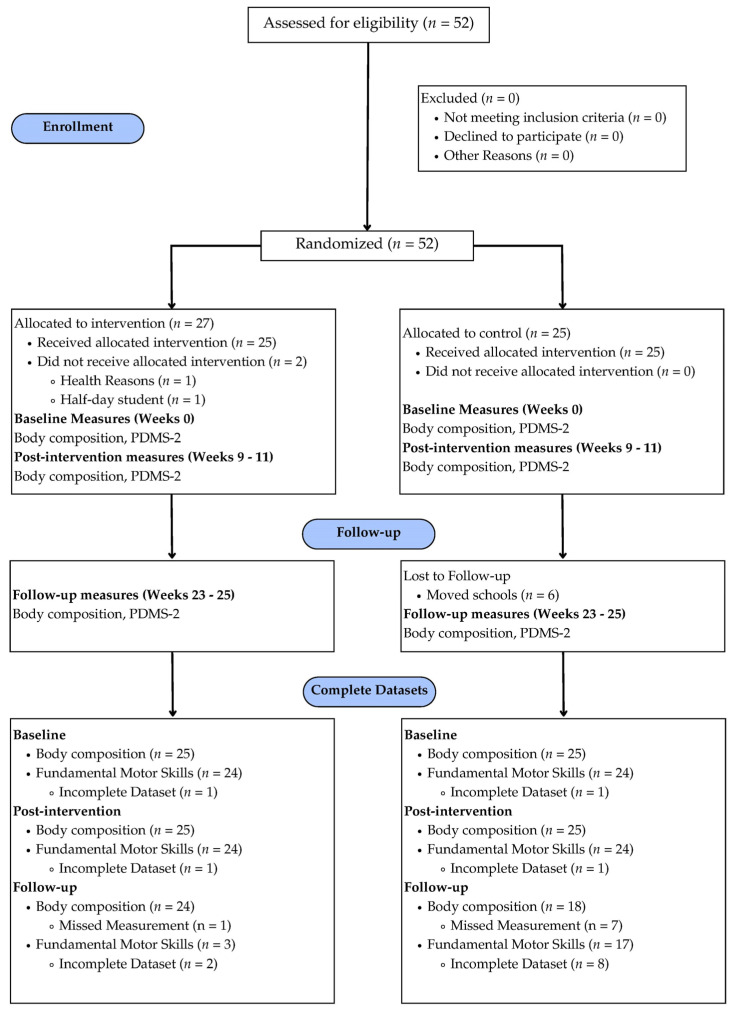
CONSORT flow diagram.

Prior to any assessments, centers were randomly assigned to the intervention or control group using a random number generator. A CONSORT flow diagram can be found in Figure 1. Baseline measures were assessed from 23 August to 10 September 2021 (week 0). During this time, teachers in the intervention group were provided with an active play resource kit. After the 8 weeks, researchers assessed post-intervention measures (weeks 9–11). To note, the following week was Thanksgiving break, and there was no school. During weeks 13–15, teachers in the intervention group were encouraged to continue the intervention. Winter break took place from week 16 to week 18, and following this time, teachers in the intervention group were encouraged to continue the intervention for the remaining school year and provided additional activities that corresponded with their weekly learning objectives (weeks 18–29). In March 2022, researchers assessed follow-up measures (weeks 30–33). Researchers measured height, weight, body composition, and FMSs at each assessment point. Due to COVID-19 protocols, researchers were limited to 15 min time frames when interacting with participants. To adhere to this protocol, a timer was started each time a new participant came into the assessment room. When the timer went off, the first author returned the participant to the classroom and pulled the next participant. It took approximately three 15 min sessions to assess all measures for each participant. All measures are discussed in detail below.

#### 2.1.2. Demographics

Biological sex, birthdate, and race were obtained from the preschool center and parents at the beginning of data collection. Additionally, researchers recorded which participants participated in extra movement opportunities offered by the childcare center. There were three main extra movement opportunities offered: a sports and fitness program, a martial arts–based activity program, and a dance program. The sports and fitness program cost USD 40.00 monthly and met for 30 min weekly. The martial arts–based activity program cost USD 65.00 monthly and met for 30 min weekly. The dance program cost USD 65.00 per student or USD 85.00 per family, plus an additional USD 30.00 program fee for each student. The dance program met for 45 min each week. These three opportunities were available at both centers and conducted during school hours.

#### 2.1.3. Height, Weight, and BMI

Height was assessed according to a standard procedure [28]. Participants stood erect, without shoes, with their head in the Frankfort horizontal plane. A portable stadiometer (SECA, Hamburg, Germany) was used to measure height. Weight was assessed with a digital scale (D.C. 430-U, Tanita Corporation, Tokyo, Japan). BMI was calculated, and obesity status was determined using age-and-gender-specific growth charts [29].

#### 2.1.4. Body Composition

Body composition was assessed by foot-to-foot bioelectrical impedance (BIA) simultaneously with weight using a Tanita body composition analyzer (D.C. 430-U, Tanita Corporation, Tokyo, Japan). This scale has a recommended minimum age of 5 years; however, previous work has measured fat mass (FM), fat-free mass (FFM), and fat percentage in preschool-age children (2–5 years) using similar Tanita scales (i.e., TBF-410GS [30] and SC-331S [31]). Measurements were conducted early in the morning following breakfast. When collecting the measurement, the child was barefoot and standing upright. This foot-to-foot method of BIA is a reliable and accurate tool for the measurement of body composition in the pediatric population, with significant correlations between BIA and dual-energy X-ray absorptiometry (DEXA) for FM (*r* = 0.98) and FFM (*r* = 0.98) [32]. The outcome measures from the data collected via BIA were FM and FFM.

#### 2.1.5. Fundamental Motor Skills

Fundamental motor skills were assessed with the Peabody Developmental Motor Scales, Second Edition (PDMS-2). This assessment is a norm- and criterion-referenced fine and gross motor skill test designed for children from birth through age 5 years and 11 months. Reliability and validity are reported in the test manual with high coefficients for content sampling (0.89–0.96), time sampling (0.89–0.94), and interrater reliability (0.89–0.96) [33]. Furthermore, content validity has been determined to be satisfactory [33]. Testing time of the three subscales (i.e., locomotion, object manipulation, and stationarity) has been reported to take about 20–25 min to administer [34]. In the present study, The PDMS-2 took the first author approximately two 15 min sessions, or 30 min in total.

The PDMS-2 consists of six subsections—two fine motor assessments (grasping and visual-motor integration) and four gross motor assessments (i.e., reflexes, stationary, locomotor, and object manipulation). Testing for each child is individualized and specifically based on developmental age–based milestones for five subsections (i.e., grasping, visual-motor integration, stationary, locomotor, and object manipulation). Thus, only the developmentally appropriate gross motor assessments for our sample were administered for this study, which included stationary skills (SS), locomotor skills (LS), and object manipulation skills (OMS). The stationary skills subsection consists of 30 items designed to test a child’s ability to control their body and maintain balance. Some example test items include standing on one foot and standing on tiptoes. The locomotor skills subsection consists of 89 items that measure a child’s ability to move from one place to another by crawling, walking, running, hopping, or jumping. Finally, the object manipulation skills sub-section consists of 26 items designed to assess a child’s ability to manipulate balls. Some example test items include catching, kicking, and throwing balls.

Scores for each task were scored on three levels of performance: 2 = performed the task correctly, 1 = performed tasks partially, and 0 = did not execute the developmental criteria correctly. The sum of the points comprises the raw scores for each subscale [33]. The raw score ranges for each subscale were 6–60 for stationary skills, 6–178 for locomotion skills, and 6–48 for object manipulation skills. Using age-appropriate tables in the testing manual [33], researchers converted the raw scores to standardized scores (0–20) and summed them to determine the gross motor quartile (GMQ), which included a range of 41–164.

### 2.2. Experimental Conditions

During a typical day, both schools had a scheduled 15 min structured physical activity indoors and two 30 min of outdoor times; one in the morning and one in the afternoon if the children were present. The intervention was designed to add active play opportunities to the day, while allowing both the intervention and control group to continue with their typically scheduled activities.

#### 2.2.1. Intervention

The 8-week active play intervention consisted of two additional teacher-guided play opportunities daily. In addition to the already scheduled structured physical activity mentioned previously, the children in the intervention group participated in an additional 15-min indoor and 15-min outdoor teacher-guided active play activity. Teachers were provided a binder with resources from *Munch & Moves* (reproduced by permission, NSW Health © 2020) in which a detailed explanation of the program can be found in Hardy and colleagues’ [23] evaluation. The present study only implemented the active play activities. These activities were designed to stimulate active play by incorporating FMS activities associated with an animal character. For example, jumping was associated with “Franky the Frog”, and one of the popular games was “Can You Cross the River”? This game would instruct children to form a line behind a drawn “river”, followed by the teacher demonstrating how to jump across the “river”. As children improved their jumping skills, teachers could extend the width of the “river” to increase the challenge. In addition to the animal-based activities, supplementary active play opportunities could be implemented indoors, incorporating locomotor and object manipulation skills. For example, one activity was “Move Like A…”, in which teachers would set up various animal pictures throughout the room. Each child would take turns picking an animal, and the class would move like the selected animal. For example, if Timmy selected a horse, the teacher would state, “Timmy picked a horse. Let’s all gallop like a horse”. Then, the teacher would demonstrate how to move around the room like a horse. Each teacher-guided activity was designed to incorporate a short warm-up, 10 min of the activity, followed by a cool down. Following the 15 min outdoor active play activity, teachers were given the option to allow children to participate in child-directed play, free-play, or continue the active play activity for the remaining time outdoors. Meaning that children in the intervention group had autonomy to interact with the playground equipment or other developmentally appropriate activities or continue the active play activity.

In addition to a binder of active play examples, teachers in the intervention group received a play equipment kit. This kit contained small equipment, such as small balls, scarves, bean bags, kick balls, polyspots, and FMSs cue books to assist teachers in implementing active play activities. The FMSs cue books were a modified version of the Fundamental Movement Skills in Action, a *Munch & Move* resource. These cue books provided teachers with images of children completing FMSs, as well as cues on how to encourage the proper movement form and technique. Teachers were also asked to implement the activities at the same time each day to achieve consistent implementation of the intervention. At the end of the 8-week intervention, teachers were encouraged to continue implementing both daily active play opportunities during the spring semester.

Before starting the intervention, the lead author spoke with each teacher to ensure comfortability in implementing the activities, as well as address any questions. Formal teacher training was approximately 60 min per teacher over the course of the intervention. This training consisted of a description of the intervention, the goals of the intervention, and the fundamentals of active play. Moreover, there was an emphasis placed on the times and locations when and where the activities could be consistently implemented each day to increase habit formation and consistent implementation. Following the training, the first author and each classroom’s teacher discussed implementation practices. All teachers reported feeling comfortable with implementing the indoor activities but asked for assistance with implementing the outdoor activities following training. Therefore, a train-the-teacher model was utilized for outdoor activities. Throughout the first 2 weeks of the intervention, teachers implemented all indoor activities, and the research group (consisting of the first author and undergraduate researchers) implemented 15 min researcher-led outdoor active play opportunities. During weeks 3 and 4, only the first author was present to assist the teachers with implementing the outdoor activities. All teachers conducted indoor and outdoor activities independently during the last 4 weeks of the 8-week intervention. At the end of each week, the first author met with lead teachers in each classroom to discuss how the implementation went that week and to troubleshoot any barriers. These discussions lasted 3–5 min.

#### 2.2.2. Control

Classes assigned to the control group continued with their typical schedule. Children in the control group continued participating in outdoor play and the scheduled, structured indoor physical activity. However, they did not participate in the additional teacher-guided active play opportunities (indoors or outdoors), and teachers did not have access to the intervention materials until the completion of follow-up analyses.

### 2.3. Data Analysis

This study was originally designed and powered to be analyzed using repeated measures ANOVAs. Due to missing data, the first author initially ran two separate repeated measures ANOVAs—one looking at baseline to post-intervention, and the second baseline, post-intervention, and follow-up. When conducting preliminary statistics to ensure that data met all the test assumptions, it was determined that for the FMS data, there was no homogeneity of covariance according to Box’s (*p* < 0.001). As a result, authors altered the analysis to a linear mixed effects regression. RStudio (version 2022.07.2) and R (version 4.3.2) were used to run linear mixed effects regression (LMER) tests using the lme4 and lmerTest packages [35,36]. The primary model of interest included fixed effects of Group, Time, as well as a random intercept to control for within-subject variance. For all analyses, pairwise comparisons of the estimated marginal means (EMMs) using the emmeans package [37] from the LMER models were used to decompose significant main effects and interactions (with a Tukey’s adjustment for multiple comparisons). In an effort to guide future research, exploratory analyses were run to evaluate nonsignificant main effects and interactions for a priori hypothesized comparisons. The level of significance was set to <0.05 for all analyses.

## 3. Results

The average age of our participants at baseline was 3.8 ± 0.7 years. The baseline sample was predominantly female (54%) and Caucasian (78%). Our racial breakdown at baseline was 78% Caucasian, 10% Black, 4% Asian, and 8% Other. Despite being offered additional movement opportunities, the majority (42%) of the sample did not participate in extracurricular activities offered by the school. This was followed closely by 30% who participated in the sports and fitness program, 18% who participated in dance, 6% who participated in the martial arts–based activity program, and 4% who participated in the sport and fitness program as well as the martial arts program. At baseline, most of the children a had a healthy weight (82%), 10% of the sample consisted of children with obesity or were overweight, and 4% of children were considered underweight based on BMI percentile classifications. Full breakdown of demographic characteristics can be found in Table 1. Most of our participants (62.5%) were classified as having average motor skill scores at baseline (Table S1: FMS Classifications).

### 3.1. Fundamental Motor Skills

**Figure 2:**
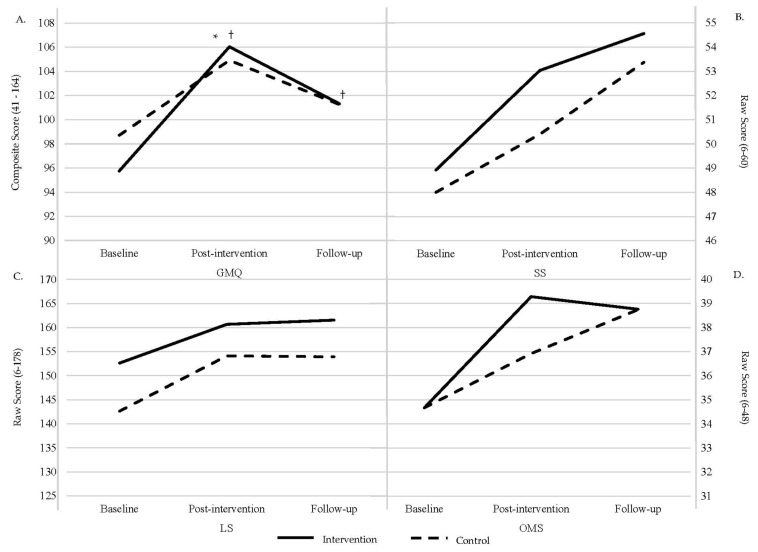
Changes in FMSs across time. GMQ is displayed in (**A**); SS in (**B**); LS in (**C**); and OMS in (**D**). † significant effect of time. * Significant interaction.

An LMER was run for GMQ but due to the small sample size, we did not have the power to run LMER for the FMS subsections (SS, LS, OMS). Table 2 displays the estimated marginal means (EMM), standard error (SE), degrees of freedom (df), and 95% confidence interval (95% CI) for each group (intervention, control) at each time point (baseline, post-intervention, and follow-up). Figure 2 displays changes in FMSs across time.

The primary LMER model included Group, Time, and their interaction as fixed effects and a random intercept to control for within-subject variance. There was a significant Group × Time interaction (β = −4.47, *t*(2, 89.44) = −2.18, *p* = 0.03) at post-intervention and main effect of Time at both post-intervention (β = 10.35, *t*(2, 89.44) = 7.15, *p* < 0.001) and follow-up (β = 5.62, *t*(2, 89.70) = 3.82, *p* < 0.001), but no main effect of Group (β = 3.35, *t*(1, 69.01) = 1.14, *p* = 0.26). Post hoc analyses revealed that the intervention group showed higher GMQ scores at post-intervention (*t*(2, 94.4) = −10.35, SE = 1.48, *p* < 0.001) compared to the control group; however both groups exhibited significant improvements in GMQ scores from baseline to post-intervention (*t*(2, 94.4) = −7.75, SE = 1.05, *p* < 0.001), post-intervention to follow-up (*t*(2, 95.7) = −3.67, SE = 1.13, *p* = 0.004), and baseline to follow-up (*t*(2, 95.3) = 4.44, SE = 1.11, *p* = 0.004).

Exploratory analyses evaluated the nonsignificant Group × Time interaction at follow-up (β = −3.89, *t*(2, 90.68) = −1.77, *p* = 0.08) and main effect of Group (β = 3.35, *t*(1, 69.01) = 1.14, *p* = 0.26). Follow-up post hoc pairwise comparisons of the estimated marginal means did indeed show no significant Group × Time interaction at follow-up (*p* > 0.05). Additionally, regarding the main effect of Group, while the control group had higher GMQ scores than the intervention group when compared across the intervention (*t*(1, 52.3) = −0.56, SE = 2.75, *p* = 0.84), this seems to have been driven by the control group having higher baseline scores compared to the intervention group.

### 3.2. Body Composition

**Figure 3:**
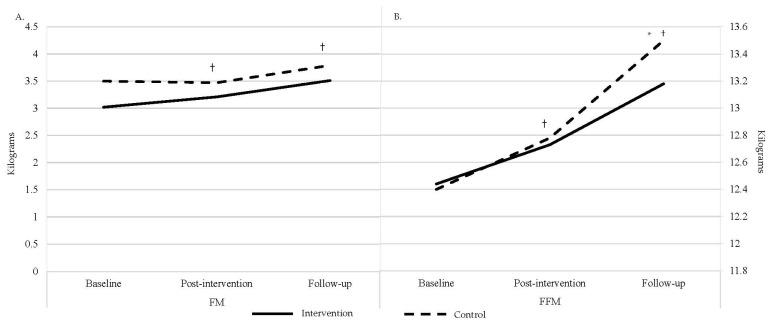
Changes in body composition across time. FM is displayed in (**A**); FFM is displayed in (**B**). † Significant effect of time. * Significant interaction.

Separate LMER tests were run for FM and FFM. Table 3 displays the estimated marginal means, standard error, degrees of freedom, and 95% confidence interval for each group (intervention and control) at each time point (baseline, post-intervention, and follow-up). Figure 3 displays changes in FM and FFM across time.

#### 3.2.1. FM

The primary LMER model for FM included Group, Time, and their interaction as fixed effects and a random intercept to control for within-subject variance. There was no significant Group × Time interaction (*p* > 0.05). However, there was a significant main effect of Time at both post-intervention (β = 0.18, *t*(2, 92.13) = 2.05, *p* = 0.04) and follow-up (β = 0.49, *t*(1, 92.27) = 5.38, *p* < 0.001), with no significant main effect of Group (β = 0.47, *t*(1, 60.33) = 1.97, *p* = 0.05). Post hoc analyses revealed a significant change in FM from baseline to follow-up (*t*(2, 96.8) = −0.39, SE = 0.07, *p* < 0.001) and post-intervention to follow-up (*t*(2, 96.8) = −0.31, SE = 0.07, *p* = 0.001). These results indicate that there was a significant increase in body fat across both the control and intervention groups from baseline to post-intervention and post-intervention to follow-up, but not baseline to follow-up.

Exploratory analyses for the Group × Time interactions indeed revealed no significant Group × Time interaction (*p* < 0.05). For the Group main effect, when compared across the intervention, the control group had higher FM (*t*(1, 52.2) = −0.33, SE = 0.23, *p* = 0.16), which similar to GMQ scores, appears to be driven by the control group having higher FM at both baseline and post-intervention compared to the intervention group.

#### 3.2.2. FFM

The primary LMER model for FFM included Group, Time, and their interaction as fixed effects and a random intercept to control for within-subject variance. There was a significant Group × Time interaction for the control group at follow-up (β = 0.294, *t*(1, 92.2) = 2.02, *p* < 0.05) and a significant main effect of Time at both post-intervention (β = 0.10, *t*(2, 92.01) = 3.04, *p* = 0.003) and follow-up (β = 0.76, *t*(2, 92.05) = 7.82, *p* < 0.001). There was no significant main effect of Group (β = 0.44, *t*(1, 53.10) = −0.10, *p* = 0.92). Post hoc analyses indeed revealed significant Group × Time interaction for FFM (*t*(2, 96.2) = −0.39, SE = 0.10, *p* = 0.002) such that the control group exhibited higher FFM at follow-up compared to the intervention group. Additionally, there was a significant change in fat-free mass over time from baseline to post-intervention (*t*(2, 96.2) = −0.34, SE = 0.07, *p* < 0.001), post-intervention to follow-up (*t*(2, 96.4) = −0.90, SE = 0.07, *p* < 0.001), and baseline to follow-up (*t*(2, 96.4) = −0.57, SE = 0.07, *p* < 0.001) for the both the control and intervention groups.

Exploratory analyses indicated that the control group had overall higher FFM than the intervention group (*t*(1,52.1) = −0.09, SE = 0.44, *p =* 0.84), which can be seen in Figure 3 below. In regard to the Group × Time interaction at post-intervention (β = 0.10, *t*(2, 92.01) = 0.71, *p* = 0.48), the pairwise comparison showed that the control group exhibited a higher FFM at post-intervention compared to the intervention group, but this failed to reach significance.

## 4. Discussion

To our knowledge, this was one of the first active play interventions in the United States that examined the effects of active play on body composition and FMSs. Findings from the current study partially support our hypotheses. Results indicate that children who received the 8-week active play intervention had significant improvements in their GMQ at post-intervention compared to the control group. Nineteen weeks following the intervention, the intervention group did see a decrease in their GMQ scores, and there was no longer a significant interaction or group differences. Concerning body composition, interestingly, the control group saw significant increases in FFM compared to the intervention group.

The current study’s results indicate that active play interventions might be a successful pathway to improve FMSs. Our findings support those of other active play studies, which have found that guided active play can result in improved FMS. In particular, the current study further indicates that the *Munch & Move* active play activities are successful at improving FMS. Hardy et al. [23] evaluated the effectiveness of the *Munch & Move* health intuitive in 28 Australian preschoolers who were included in a randomized control trial. The study required school staff in the intervention group to attend a full-day professional development workshop. Moreover, the researchers in Hardy et al. [23] provided the preschools with a manual and a small grant to support staff training or equipment purchasing. Additionally, contact with a local health promotion professional to support the program delivery was provided [19]. At the completion of 4 months, Hardy and colleagues [23] found that the intervention significantly improved locomotor, object control, and total FMSs. In comparison, our results showed the intervention significantly improved GMQ across 8 weeks by 10.75%; however, at follow-up testing, the intervention group showed a 5.17% increase from baseline compared to the control group who showed a 2.34% increase. There are some noteworthy differences between the two studies. First, we utilized the PDMS-2, whereas Hardy et al. [23] utilized the Test of Gross Motor Development, Second Edition (TGMD-2). Secondly, training time and resources given to teachers in our study was less. Despite these differences, our 8-week active play intervention was successful at improving GMQ acutely with only 60 min of training per teacher and limited support from the research team. Furthermore, while there were no significant group differences, at follow-up, the intervention group did have higher GMQ scores than their baseline, indicating that the 8-week active play intervention significantly improved GMQ acutely, and that these benefits were still observed 19 weeks after the intervention.

While the TGMD-2 is most commonly used to assess motor skills in active play interventions, the PDMS-2 is considered an efficient outcome measure for evaluating motor improvement and overall developmental changes in children [38]. The PDMS-2 was originally chosen as the motor assessment for the current study because we intended to assess fine and gross motor skills. Unfortunately, due to COVID-19 restrictions, we were unable to assess fine motor skills. Regardless, our findings are like other preschool movement interventions that utilize the PDMS-2. In a 12-week pilot study, Biino et al. [39] found that a cognitively engaging physical activity intervention, similar to guided active play, resulted in significant improvement in PDMS-2 gross motor skills. Thus indicating that regardless of process evaluation, the overall trend indicates that active play interventions can improve fundamental motor skills in preschoolers [21,22,23,39], and school-aged children [40].

Active play interventions, which are still relatively understudied, have primarily focused on changes to physical activity or FMSs. We could only identify one other study that included body composition. Goldfield and colleagues [24] found that a 6-month active play intervention was effective at decreasing body fat percentage and FM in the intervention group; however, there were no significant differences between groups for FFM, BMI, or z-BMI. The results of the current study do not support those of Goldfield [24]. However, previous research from our lab has found that an FMS intervention might effectively reduce the risk of increasing FM in low-income preschoolers [41]. Nonetheless, the current active play intervention did not show similar results. The difference in our intervention’s length and/or total dose may be a contributing factor [24,41]. To accommodate the preschool center’s schedule, our study was implemented for 8 weeks instead of 6 months [24] or 9 months [41]. It is possible that the timing of our intervention did not allow enough time to observe any physiological differences in body composition. Current research suggest that childhood obesity is a public health crisis, with one in three children aged 2–5 years classified as obese [42]. Moreover, Lagstrom and colleagues found that weight gain at 2–3 years predicted weight status at 13 years [33]. For this study, participants increased FM and FFM by approximately half a pound. Changes in body composition in preschoolers are still not fully understood, and more research is needed to determine how preschools can contribute to a healthy weight and what changes constitute developmentally appropriate changes. Future research would benefit from observing the changes in body composition across the school year. Furthermore, it would be beneficial to examine relationships between body composition and FMSs because current literature indicates that FMSs might be deficit in obese children [43].

Though not included in this analysis, teachers on average implemented active play opportunities 56% of possible times indoors and 65% of possible times outdoors across all the classrooms. Anecdotally, researchers noted that there was a big push to focus on academic material at the preschool centers during the intervention. Several teachers cited this as the reason for not fully implementing the intervention. Preschool teachers often share these views because they continue to have increased academic demands [44]. During the follow-up time period, we were unable to fully document active play implementation, but teachers verbally reported that they implemented the indoor active play activities as an indoor movement activity a few times a week. However, the teachers did not implement the outdoor active play activities and opted for free play during outdoor time. Teachers may require more training to implement outdoor FMS activities, and/or may perceive their role as more of an observer or onlooker versus an involved teaching role during outdoor play [45]. In the future, it would be beneficial for researchers and educators to work together on interventions that might be less burdensome by incorporating play and fundamental motor skills into the curriculum. Moreover, most of the sample was classified as healthy weight (intervention = 92%, control = 80%), 66.65% of the sample was classified as average, and 14.6% was classified as above average for baseline GMQ. While having children with a healthy weight or competent in motor skills is a global goal, these baseline values could affect the study results. Future research would benefit from reproducing this intervention in more at-risk preschools to determine if this intervention is appropriate and successful for all preschoolers.

### Limitations

While this study aimed to fill in current literature gaps, it has limitations. One limitation of this study is that BIA measurements took place after breakfast. Few studies have measured BIA in children. However, adult guidelines stipulate that individuals should be 4–8 h fasted [46,47]. Research has found that measuring 2–4 h after a meal overestimates FFM [48]. While it is acceptable to follow these guidelines with children, there is an ethical question of preventing a child from eating when you are unsure of their last meal [49]. While we acknowledge that it is a limitation to measure following a meal, BIA in the current study was measured within 1 h of eating. Furthermore, all BIA measurements occurred following breakfast at baseline, post-intervention, and follow-up, thus attempting to control for any variability. limitation of this study is the failure to measure potential home environment impacts, such as extracurricular activities, diet, or sleep. While these factors are outside the scope of the current study, we acknowledge that they can influence our measures.

Finally, there is a minor possibility that the weather impacted our findings. The 8-week intervention took place from 13 September 2021–5 November 2021. Post-intervention assessments took place 8–12 November 2021, and follow-up assessments took place in March 2022. Meaning that the intervention and all measures took place from late fall to winter. While this is often a time marked by lower temperatures, thereby limiting the ability to engage in outdoor play, this study did take place in the Southeastern region of the United States. During the intervention period, the average temperature was 69 °F (~21 °C), and throughout the entire study, the average temperature was about 58 °F (~14 °C).

## 5. Conclusions

Results of the current study indicate that active play interventions might be a possible pathway to promote FMSs. While we did not find any significant intervention effects on body composition, it is possible that through active play, children can learn better health behaviors that in turn can lead to impacts later in life. Future studies would benefit from observing the changes in body composition, FMSs, and active play throughout the year, because little is understood about these relationships in early childhood. Furthermore, it would be prudent to create a teacher training designed to inform teachers about the importance of active play, train them in how to implement active play, and provide them with the tools to develop healthy behaviors. 

## Figures and Tables

**Table 1:** Demographic variables.

	Baseline		Post-Intervention		Follow-Up	
	Intervention (*n* = 25)	Control (*n* = 25)	Intervention (*n* = 25)	Control (*n* = 25)	Intervention (*n* = 24)	Control (*n* = 18)
**Age (years)**	3.91 (±0.53) [3.69–4.14]	3.69 (±0.81) [3.35–4.03]	4.13 (±0.54) [3.90–4.36]	3.86 (±0.81) [3.52–4.20]	4.47 (±0.59) [4.23–4.72]	4.27 (±0.88) [3.83–4.70]
**Height (cm)**	101.14 (±5.88) [98.77–103.52]	99.62 (±5.94) [97.24–101.99]	102.57 (±5.63) [1.41%] [100.22–104.92]	101.09 (±6.05) [1.48%] [98.74–103.44]	103.94 (±6.27) [2.77%] [101.50–106.38]	104.01 (±5.73) [4.41%] [101.21 = 106.80]
**Weight (kg)**	15.46 (±1.96) [14.59–16.34]	15.89 kg (±2.38) [15.02–16.77]	15.94 (±2.00) [3.10%] [15.04–16.84]	16.26 (±2.46) [2.33%] [15.36–17.16]	16.70 (±2.2) [8.02%] [15.76–17.65]	17.14 (±7.84) [7.87%] [16.08–18.49]
**BMI (kg/m^2^)**	15.08 (±0.88) [14.66–15.50]	15.95 (±1.19) [15.53–16.37]	15.12 (±0.99) [0.27%] [14.70–15.54]	15.84 (±1.10) [−0.69%] [15.42–16.37]	15.43(±1.24) [2.32%] [14.96–15.91]	15.92 (±1.08) [−0.19%] [15.38–16.46]
**BMI%**	37.12 (±23.46%) [26.41–47.83]	53.56 (±29.48%) [42.85–64.27]	39.04 (±24.99) [5.17%] [27.99–50.08]	53.24 (±29.72) [−0.60%] [42.20–64.28]	47.14 (±28.42) [26.99%] [36.10–58.22]	58.10 (±25.99) [8.48%] [45.42–70.79]
**F%**	19.36 (±4.5%) [17.84–20.88]	21.64 (±2.85%) [20.12–23.16]	20.06 (±3.22) [3.62%] [18.88–21.25]	21.17 (±2.64) [−2.17%] [19.99–22.36]	20.94 (±3.31) [8.16%] [19.78–22.11]	21.67 (±2.21) [0.14%] [20.34–23.01]
**FM (kg)**	3.03 (±0.92) [2.66–3.39]	3.50 (±0.91) [3.13–3.87]	3.21 (±0.76) [5.94%] [2.88–3.54]	3.47 (±0.87) [−0.86%] [3.15–3.80]	3.51 (±0.89) [15.84%] [3.14–3.87]	3.74 (±0.86) [6.86%] [3.32–4.16]
**FFM (kg)**	12.44 (±1.37) [11.84–13.04]	12.40 (±1.60) [11.80 = 12.99]	12.72 (±1.51) [2.25%] [12.09–13.38]	12.78 (±1.70) [3.06%] [12.13–13.43]	13.19 (±1.65) [6.03%] [12.50–13.88]	13.44 (±1.73) [8.39%] [12.64–14.24]
	Intervention (*n* = 24)	Control (*n* = 24)	Intervention (*n* = 24)	Control (*n* = 24)	Intervention (*n* = 23)	Control (*n* = 17)
**SS**	48.96 (±5.90) [46.51–51.32]	48.00 (±5.8) [45.60–50.40]	53.04 (±4.27) [8.33%] [51.56–55.11]	50.40 (±4.48) [5.00%] [48.72–52.28]	54.52 (±3.37) [11.36%] [53.20–55.85]	53.59 (±2.79) [11.65%] [52.05–55.13]
**LS**	152.63 (±16.28) [141.36–163.89]	137.71 (±35.19) [126.44–148.97]	160.68 (±17.77) [5.27%] [155.28–169.06]	154.12 (±18.06) [11.92%] [148.97–162.29]	162.30 (±15.63) [6.34%] [154.05–170.56]	154.18 (±23.93) [11.96%] [144.57–164.63]
**OMS**	34.67 (±6.48) [31.97–37.37]	34.67 (±6.66) [31.97–37.37]	39.28 (±4.63) [13.30%] [37.40–41.52]	36.92 (±5.40) [6.49%] [35.10–39.23]	39.13 (±4.91) [12.86%] [37.19–41.07]	39.22 (±4.54) [13.12%] [37.10–41.61]
**GMQ**	95.75 (±12.28) [90.99–100.51]	98.71 (±10.87) [93.95–103.47]	106.04 (±9.41) [10.75%] [101.86–110.23]	104. 93 (±23.36) [6.30%] [100.40 = 108.77]	100.70 (±8.33) [5.17%] [96.85–104.54]	101.00 (±10.10) [2.34%] [96.53–105.48]

Notes: Mean (S.D.) [% change from baseline], [95% confidence interval], body mass index (BMI), body mass index percentile (BMI%), body fat percentage (F%), fat mass (FM), fat-free mass (FFM), stationary skills (SS), locomotor skills (LS), and object manipulation skills (OMS) are the mean of raw scores, which are then used to calculate gross motor quartile (GMQ). There is no unit of measure for these four items.

**Table 2:** GMQ Group × Time estimated marginal means.

		EMM	df	SE	95% CI
Baseline	Intervention	−5.55	72.8	2.12	[−9.78, −1.31]
	Control	−2.20	72.8	2.11	[−6.43, 2.04]
Post-Intervention	Intervention	4.80	71.1	2.12	[0.60, 9.01]
	Control	3.68	71.1	2.12	[−0.52, 7.89]
Follow-up	Intervention	0.07	72.8	2.11	[−4.16, 4.3]
	Control	−0.47	86.5	2.25	[−4.93, 4.00]

**Table 3:** Body Composition Group × Time estimated marginal means.

			EMM	df	SE	95% CI
**Baseline**	**Intervention**	FM	−0.37	62.7	0.17	[−0.071, −0.02]
		FFM	−0.36	55.3	0.32	[−0.99, 0.28]
	**Control**	FM	0.10	62.7	0.17	[−0.24, 0.45]
		FFM	−0.40	55.3	0.32	[−1.04, 0.24]
**Post-Intervention**	**Intervention**	FM	−0.18	62.7	0.17	[−0.53, 0.16]
		FFM	−0.07	55.3	0.32	[−0.70, 0.57]
	**Control**	FM	0.08	62.7	0.17	[−0.26, 0.43]
		FFM	−0.14	55.3	0.32	[−0.65, 0.62]
**Follow-up**	**Intervention**	FM	0.13	63.7	0.17	[−0.22, 0.47]
		FFM	0.40	55.7	0.32	[−0.24, 1.04]
	**Control**	FM	0.39	72.0	0.18	[0.03, 0.75]
		FFM	0.65	58.3	0.32	[0.01, 1.29]

## Data Availability

The data sets generated and analyzed during the current study are not publicly available due to a lack of informed consent for data sharing. However, they are available from the corresponding author upon reasonable request by email.

## Supplementary Materials

The following supporting information can be downloaded at: https://www.mdpi.com/article/10.3390/children11101173/s1, Table S1: FMS Classifications.
