# Supplementary material for: Effects of an 8-Week Active Play Intervention on Body Composition and Fundamental Motor Skills in Preschool Children
Source: Children (Basel). 2024 Sep 26;11(10):1173. doi: 10.3390/children11101173 (PMC11506747; doi:10.3390/children11101173)
Supplement: Supplementary file 1 [file children-11-01173-s001.zip › children-3218504-supplementary.pdf]

Supplementary Table S1. FMS Classifications

		Baseline		Post-intervention		Retention	
		Intervention	*Control*	*Intervention*	*Control*	*Intervention*	*Control*
**SS**	***Below Average***	29.2 %	16.7 %	-	8.3 %	-	-
	Average	45.2 %	66.7 %	70.8 %	70.8 %	88.3 %	66.7 %
	Above Average	16.7 %	16.7 %	29.2 %	20.8 %	16.7 %	33.3 %
**LS**	***Below Average***	20.8 %	8.3 %	8.3 %	8.3 %	16.7 %	11.1 %
	Average	70.8 %	87.5 %	50.0 %	70.8 %	62.5 %	83.3 %
	Above Average	8.3 %	4.2 %	41.7 %	20.3 %	20.8 %	5.6 %
**OMS**	***Below Average***	41.7 %	25.0 %	4.2 %	12.5 %	20.8 %	16.7 %
	Average	54.2 %	66.6 %	87.5 %	79.2 %	75.0 %	77.8 %
	Above Average	4.2 %	8.3 %	8.3 %	8.3 %	4.2 %	5.5 %
**GMQ**	***Below Average***	25.0 %	12.5 %	8.3 %	8.3 %	8.3 %	11.1 %
	Average	62.5 %	70.8 %	58.3 %	54.2 %	70.8 %	72.2 %
	Above Average	12.5 %	16.7 %	33.3 %	37.5 %	20.8 %	16.7 %

Notes: Mean (S.D.), Stationary Skills (SS), Locomotor Skills (LS), and Object Manipulation Skills (OMS) are the mean of raw scores, which are then used to calculate Gross Motor Quartile (GMQ). There is no unit of measure for these four items.
